# Quantitative CT‐Based Methods for Bone Microstructural Measures and Their Relationships With Vertebral Fractures in a Pilot Study on Smokers

**DOI:** 10.1002/jbm4.10484

**Published:** 2021-03-19

**Authors:** Xiaoliu Zhang, Alejandro P Comellas, Elizabeth A Regan, Indranil Guha, Amal Shibli‐Rahhal, Mishaela R Rubin, Paul A DiCamillo, Elena M Letuchy, R Graham Barr, Eric A Hoffman, Punam K Saha

**Affiliations:** ^1^ Department of Electrical and Computer Engineering, College of Engineering University of Iowa Iowa City IA USA; ^2^ Department of Internal Medicine, Carver College of Medicine University of Iowa Iowa City IA USA; ^3^ Division of Rheumatology, Department of Medicine National Jewish Health Denver CO USA; ^4^ Department of Clinical Medicine Columbia University New York NY USA; ^5^ Department of Radiology, Carver College of Medicine University of Iowa Iowa City IA USA; ^6^ Department of Epidemiology, College of Public Health University of Iowa Iowa City IA USA; ^7^ Department of Medicine Columbia University New York NY USA; ^8^ Department of Biomedical Engineering, College of Engineering University of Iowa Iowa City IA USA

**Keywords:** OSTEOPOROSIS, SMOKING, VERTEBRAL FRACTURE, CORTICAL BONE, TRABECULAR BONE, BONE MICROSTRUCTURE, CT IMAGING, MORPHOMETRY

## Abstract

Osteoporosis causes fragile bone, and bone microstructural quality is a critical determinant of bone strength and fracture risk. This study pursues technical validation of novel CT‐based methods for assessment of peripheral bone microstructure together with a human pilot study examining relationships between bone microstructure and vertebral fractures in smokers. To examine the accuracy and reproducibility of the methods, repeat ultra‐high‐resolution (UHR) CT and micro‐CT scans of cadaveric ankle specimens were acquired. Thirty smokers from the University of Iowa COPDGene cohort were recruited at their 5‐year follow‐up visits. Chest CT scans, collected under the parent study, were used to assess vertebral fractures. UHR CT scans of distal tibia were acquired for this pilot study to obtain peripheral cortical and trabecular bone (Cb and Tb) measures. UHR CT‐derived Tb measures, including volumetric bone mineral density (BMD), network area, transverse trabecular density, and mean plate width, showed high correlation (*r* > 0.901) with their micro‐CT‐derived values over small regions of interest (ROIs). Both Cb and Tb measures showed high reproducibility—intra‐class correlation (ICC) was greater than 0.99 for all Tb measures except erosion index and greater than 0.97 for all Cb measures. Female sex was associated with lower transverse Tb density (*p* < 0.1), higher Tb spacing (*p* < 0.05), and lower cortical thickness (*p* < 0.001). Participants with vertebral fractures had significantly degenerated values (*p* < 0.05) for all Tb measures except thickness. There were no statistically significant differences for Cb measures between non‐fracture and fracture groups. Vertebral fracture‐group differences of Tb measures remained significant after adjustment with chronic obstructive pulmonary disease (COPD) status. Although current smokers at baseline had more fractures—81.8% versus 63.2% for former smokers—the difference was not statistically significant. This pilot cross‐sectional human study demonstrates CT‐based peripheral bone microstructural differences among smokers with and without vertebral fractures. © 2021 The Authors. *JBMR Plus* published by Wiley Periodicals, Inc. on behalf of American Society for Bone and Mineral Research. © 2021 The Authors. *JBMR Plus* published by Wiley Periodicals LLC on behalf of American Society for Bone and Mineral Research.

## Introduction

Osteoporosis is a common age‐related disease characterized by reduced bone mineral density (BMD), microstructural deterioration, and increased fracture risk.^(^ ^1^, ^2^ ^)^ Although osteoporosis is a disease across all ages and both sexes, its prevalence increases with aging, especially among women after menopause.^(^ ^3^ ^)^ Approximately 40% of women and 13% of men suffer at least one osteoporotic fracture in their lifetime.^(^ ^4^ ^)^ Osteoporotic fractures commonly occur in the hip, spine, wrist, ankle, and upper arm. Osteoporotic hip fractures are especially devastating—20% of people who have a hip fracture die within 12 months and half of those who survive can no longer live independently.^(^ ^5^, ^6^ ^)^ Osteoporosis imaging is used to measure bone metrics with the goal to assess and monitor disease status, fracture risk, and response to treatment.^(^ ^7^ ^)^ Dual‐energy X‐ray absorptiometry (DXA)‐based areal bone mineral density (aBMD) is clinically used to define osteoporosis and fracture risk. However, by only providing two‐dimensional (2D) areal measures, DXA has several limitations, and it does not account for the impact of cortical and trabecular bone (Cb and Tb) distribution and microstructural quality on skeletal strength and fracture risk. In fact, it is generally agreed that only about 60% of the bone's mechanical competence is explained by variations in DXA‐based BMD.^(^ ^8^ ^)^ Histologic studies have convincingly demonstrated that Cb and Tb microstructural measures are critical determinants of bone strength and fracture risk.^(^ ^9^, ^10^, ^11^, ^12^, ^13^, ^14^, ^15^, ^16^, ^17^, ^18^, ^19^ ^)^

Vertebral fractures are the most common type of osteoporotic fracture in older adults,^(^ ^20^ ^)^ and prior studies suggest that only one‐fourth to one‐third of these fractures come to medical attention.^(^ ^21^, ^22^ ^)^ Vertebral fractures are associated with increased risk of back pain and functional limitations,^(^ ^23^ ^)^ and are commonly used as outcome variables in osteoporosis prevention and intervention studies.^(^ ^24^ ^)^

In the last two decades, there has been remarkable progress in high‐resolution imaging and analytic technologies, enabling in vivo assessment of trabecular bone microstructure.^(^ ^7^ ^)^ Volumetric bone imaging modalities, including magnetic resonance imaging (MRI)^(^ ^8^, ^25^, ^26^, ^27^ ^)^ and high‐resolution peripheral quantitative computed tomography (HR‐pQCT),^(^ ^28^, ^29^, ^30^ ^)^ have been investigated for quantitative assessment of bone microstructure at peripheral skeletal sites. Despite their considerable ability to produce images with high spatial resolution and provide detailed information on bone microstructure, these techniques have been limited by slow‐speed scanning, which leads to motion artifacts,^(^ ^28^ ^)^ smaller fields of view (FOV) that increase susceptibility to positioning errors,^(^ ^31^ ^)^ the need for specialized equipment and hardware, and, in the case of MRI, failure to provide quantitative BMD measures. CT‐based methods provide quantitative BMD measures and overcome the major limitations of MRI and HR‐pQCT by providing higher scan speed and larger FOV. These methods are also relatively easy to apply and calibrate for data uniformity for multisite studies.^(^ ^7^ ^)^ Additionally, emerging CT technologies generate an in vivo resolution suitable for segmentation and characterization of Cb and Tb microstructures at peripheral skeletal sites. For example, using an ultra‐high‐resolution (UHR) scan mode, the Siemens SOMATOM Force achieves 10% modulation transfer function (MTF) of 24.8 lp/cm on the *xy*‐plane and 21.0 lp/cm along the *z*‐axis, which are equivalent to 201.6 and 238.1 μm true in‐plane and *z*‐axis resolution.^(^ ^32^ ^)^ Although some studies on CT‐based methods for bone microstructural assessment are available in literature,^(^ ^32^, ^33^, ^34^, ^35^, ^36^ ^)^ thorough evaluations of the potential of CT‐based methods for bone microstructural imaging and their ability to characterize fracture risk and explain the patterns of different osteoporotic disease progression and treatment response are largely missing.

This work pursues technical validation of novel CT‐based methods for assessment of peripheral Cb and Tb microstructure on cadaveric ankle specimens together with a human pilot study examining relationships between peripheral bone microstructure and vertebral fractures in smokers.

## Materials and Methods

### Study design

Our study design includes micro‐CT and repeated CT imaging of cadaveric human ankle specimens evaluating the accuracy and reproducibility of our CT‐based methods for characterizing peripheral Cb and Tb microstructure. We also present a pilot cross‐sectional human study on smokers examining the relationships between peripheral bone microstructure and vertebral fracture prevalence. This pilot cross‐sectional study was nested in the larger COPDGene cohort study.^(^ ^37^ ^)^ Total lung capacity (TLC) chest CT scans and spirometry measures of forced expiratory volume in 1‐second (FEV1) and forced vital capacity (FVC) were collected as part of the parent COPDGene study protocol and were used in this pilot study. The FEV1 and FEV1/FVC were used to compute a GOLD classification rating as a measure of chronic obstructive pulmonary disease (COPD) status,^(^ ^38^, ^39^ ^)^ and the chest CT scan was used to identify and evaluate vertebral fractures. In addition, subjects underwent a UHR distal tibia CT scan that was used to determine peripheral Cb and Tb measurements. All data reported in this article, including distal tibia bone measures, GOLD classification, smoking history, and vertebral fracture reading, were collected at the 5‐year follow‐up visits of the COPDGene study. The study was approved by the University of Iowa Institutional Review Board, and written informed consents were collected from all participants.

### Cadaveric specimens

Fifteen fresh‐frozen cadaveric ankle specimens from 9 donors (5 females; mean ± SD of age at death 72.6 ± 5.8 years) were removed at mid‐tibia from body donors under the Deeded Bodies Program at The University of Iowa (Iowa City, IA, USA). Exclusion criteria were evidence of previous fractures or known history of bone tumor or bone metastases. These specimens were placed in a sealed plastic bag and kept frozen until CT imaging. Specimens were thawed at room temperature before scanning. Three repeat CT scans were acquired on the same day for each specimen after repositioning the specimen on the scanner table before each repeat scan. Seven specimens from 7 different donors were randomly selected for micro‐CT imaging.

### Participants

Thirty smokers were recruited from the University of Iowa cohort of the ongoing COPDGene study at their 5‐year follow‐up visits.^(^ ^37^ ^)^ Individuals with diabetes or with any cancer, except for non‐melanoma skin cancer, were not eligible for enrollment. Following the COPDGene study design, all subjects were current or former smokers with at least 10 pack‐years of smoking history. As a part of a previous study, Jaramillo and colleagues^(^ ^40^ ^)^ read chest CT scans of 561 subjects from the University of Iowa COPDGene cohort at their baseline visits and scored their morphologic vertebral fractures. This pool of subjects with known vertebral fracture status at their baseline visits was used to choose participants for the current study. Specifically, participants were enrolled on a first‐come basis to represent three equally sized groups based on their vertebral fracture status at baseline—no fracture, one fracture, and more than one fractures.

### Distal tibia UHR CT imaging

**Fig 1:**
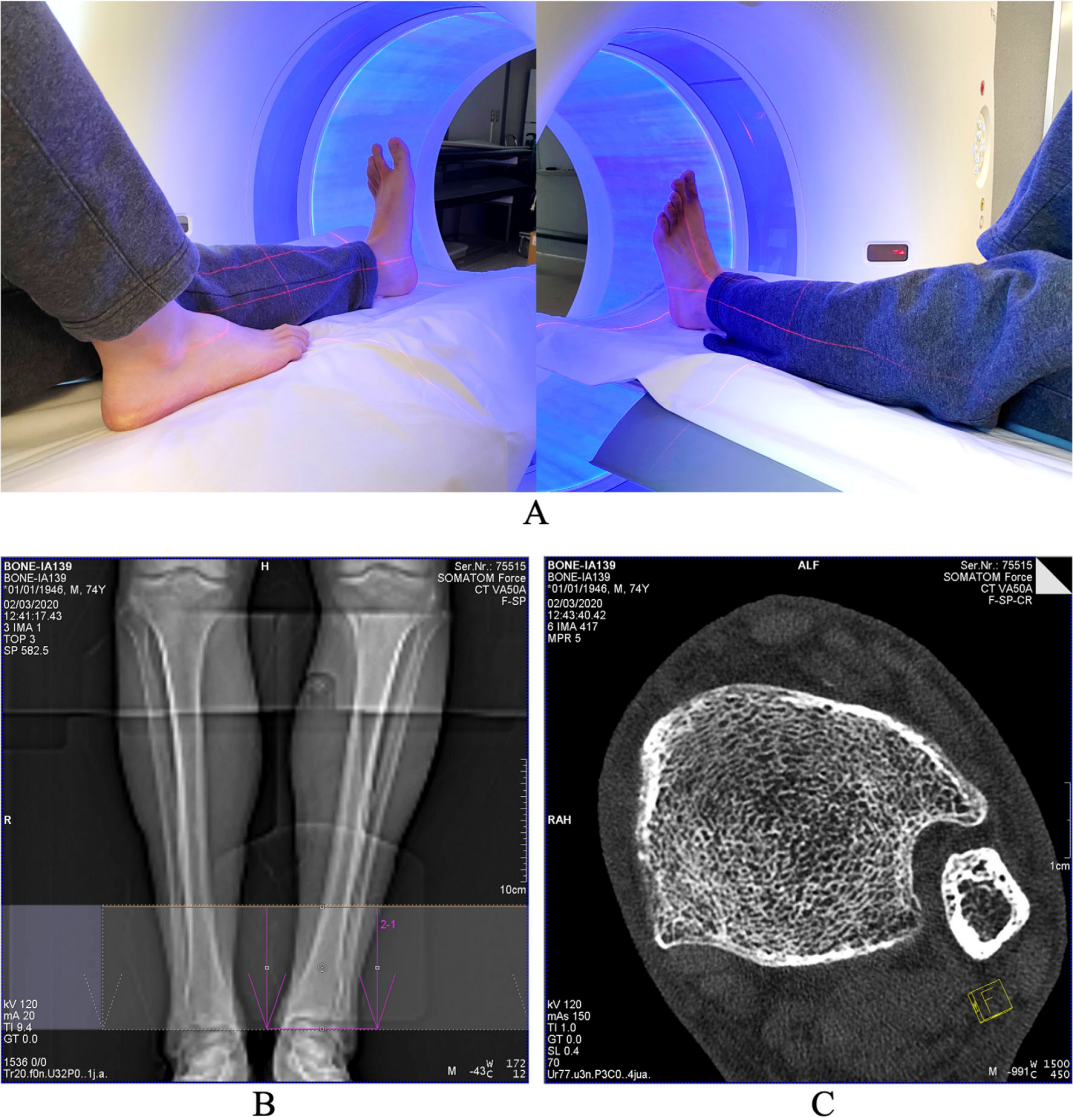
Human ankle scan setup on a Siemens FORCE CT scanner. (*A*) Positioning of the ankle. Tibial axis is aligned with the scanner center using laser rays. This alignment step is important to achieve the highest image resolution. (*B*) Positioning of the field of view (FOV) on an anterior–posterior projection CT scout scan. The distal tibial end plateau is included in the FOV, which is used to determine different tibial locations for region of interest selection during analysis. (*C*) An axial image slice from the reconstructed CT scan data.

**Fig 2:**
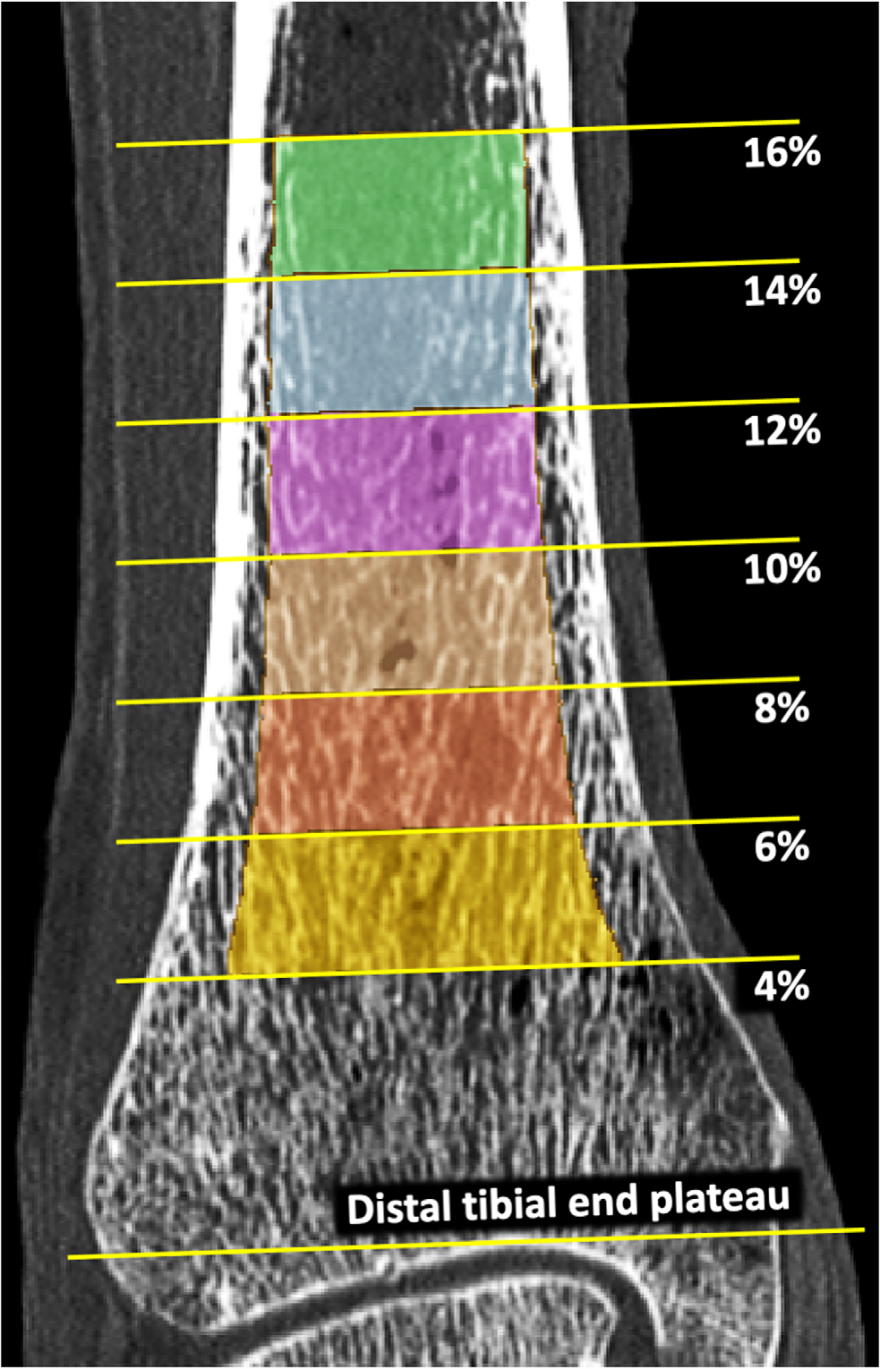
Subject‐specific regions of interest (ROIs) for cortical bone and trabecular bone measures. References of distal tibial end plateau and tibial length and percent peel approaches were used for ROI selection adjusting for size‐ and shape‐related variations among participants.

Both cadaveric and in vivo distal tibia UHR CT scans were performed on a Siemens SOMATOM Force (Forchheim, Germany) scanner located at the University of Iowa Comprehensive Lung Imaging Center (ICLIC) research CT facility using the same positioning and imaging protocols. Ankle positioning and scan setup for the Siemens FORCE CT scanner as well as the protocol for the FOV selection on a scout scan and a reconstructed axial image slice are illustrated in Fig. 1. The left leg was used for scanning unless a subject reported a prior fracture in the lower left leg, in which case the right leg was scanned. During ankle positioning, references of laser rays were used to align the tibial axis with the scanner center. Alignment of the tibial axis with the scanner center is important to achieve the highest spatial resolution for distal tibial UHR CT scans. An anterior–posterior projection CT scout scan was used to ensure the inclusion of the distal tibial end plateau in the FOV. The reference of the distal tibial end plateau was used to define different percent of tibial sites for computation of bone measures, as will be described in the next section (Fig. 2). The following CT scan parameters were used: single X‐ray source spiral acquisition at 120 kV, 100 effective mAs, 1‐second rotation speed, pitch factor 1.0, number of detector rows 64, scan time 5.8 seconds, collimation 64 × 0.6 mm, and total effective dose equivalent 50 μSv ≈ 5 days of environmental radiation in the Unites States. Siemens z‐UHR scan mode was applied enabling Siemens double z sampling technology. Images were reconstructed at in‐plane resolution of 150‐μm and 200‐μm slice spacing using Siemens's special kernel Ur77u with Edge Technology to achieve high spatial resolution. A Gammex RMI 467 Tissue Characterization Phantom (Gammex RMI, Middleton, WI, USA) was separately scanned using the same protocol to calibrate CT intensity values to BMD.

### Micro‐CT imaging of cadaveric specimens

Cadaveric specimens were scanned on Xradia 520 Versa scanner (ZEISS, Oberkochen, Germany) at the Iowa Institute for Biomedical Imaging (IIBI) laboratory after removing soft tissue and dislocating the tibia from the ankle joint. Specimens were thawed at room temperature before scanning to avoid subtle movement that can occur during thawing. The following micro‐CT parameters were used: 100 kV, 90 μA, 1601 projections over 360 degrees, exposure 4 seconds per projection, scan time 3 hours (approximately), scan length 36 mm (approximately), low‐energy filter LE4 and standard beam hardening correction were applied, and images were reconstructed using a smooth 0.5 kernel with image array size 2048 × 2048 and 18.63 μm isotropic voxel size.

### CT image processing and bone measures

Each distal tibia CT scan was visually inspected for any visible marks of motion and beam‐hardening artifacts. After clearance through the image‐quality control step, each CT scan was transformed to a BMD image at isotropic voxel resolution. CT scans of the Gammex phantom and an automated algorithm developed in our laboratory were used to calibrate CT Hounsfield numbers into BMD (mg/cc) values. After converting a CT scan to a BMD image, it was interpolated at 150 μm isotropic voxel size using a windowed sync interpolation method.^(^ ^41^ ^)^ All subsequent image processing operations were applied on BMD images at 150 μm isotropic voxel size. Micro‐CT images were converted into BMD images using a micro‐CT vendor‐supplied calibration phantom with four rod inserts with different material densities; connectivity analysis was applied to eliminate isolated noisy voxels. Finally, micro‐CT scans were downsampled at 50 μm for computational feasibility.

#### ROI selection

For the micro‐CT‐based accuracy experiment, spatially matching spherical ROIs of diameter 7.05 mm (ie, 47 voxels at 150 μm resolution) from micro‐CT and CT images were used to compute Tb measures. For each specimen, 25 ROIs were randomly selected over 4% to 8% distal tibia site with 30% peel generating a total of 175 ROIs from 7 specimens. Similar spatially matching spherical ROIs were generated in three repeat CT scans for reproducibility analysis; a total of 225 (15 × 15) ROIs were generated for the reproducibility test.

Also, a method was developed to generate axial pseudo‐cylindrical ROIs at physiologically consistent tibial sites after adjusting for subject‐specific size‐ and shape‐related variations among participants. The method works on a filled‐in tibia bone volume computed using multiscale morphology and connectivity analysis.^(^ ^42^ ^)^ Filled‐in bone was used to determine: (i) the distal tibial end plateau, (ii) the tibial axis, (iii) 2% pseudo‐cylindrical axial ROIs at specific sites of tibial length (Fig. 2), and (iv) the inner (60% peel) and outer (annular region between 30% and 60% peels) regions. The distal tibial end plateau was located just proximal to the first image slice containing a 2D hole within the filled‐in bone while tracing slices from proximal to distal direction. The 60% peel region of filled‐in bone proximal to 8% of tibial length, measured from the end plateau, was used to determine the tibial axis orientation using a mean‐square‐error line‐fitting algorithm. Pseudo‐cylindrical axial ROIs at different tibial locations (Fig. 2) were determined after aligning tibia axis with image *z*‐axis. 2D distance transform analysis of realigned filled‐in bone on individual image slices was applied to generate different percent peels. The ROIs generated using the above protocol were used for the cadaveric reproducibility (*n* = 15) as well as human study (*n* = 30) experiments.

#### Computation of bone microstructural measures

Cb and Tb measures were computed from UHR CT scans of the distal tibia. Skeletonization converts a volumetric representation to digital medial surfaces and curves,^(^ ^43^ ^)^ which is an essential preprocessing step for quantitative analysis of Tb microstructure. Because of the limited spatial resolution of the CT scanner used in the current study, individual trabeculae appeared as fuzzy microstructures in acquired images (Fig. 1*C*). To effectively handle such fuzzy representation of trabecular microstructures at limited resolution, fuzzy skeletonization^(^ ^44^ ^)^ was applied eliminating thresholding‐induced data loss associated with binarization needed for conventional skeletonization algorithms.^(^ ^43^ ^)^ CT‐derived Cb and Tb measures examined in this pilot study are described in the following paragraph.

Volumetric trabecular bone mineral density (Tb.vBMD; mg/cc) measure was computed by averaging BMD values over a target ROI, while trabecular bone network area density (Tb.NA; mm^2^/mm^3^) was computed as the number of voxels in the skeletal representation of Tb microstructure per unit volume to represent Tb network density. Tensor scale analysis^(^ ^45^ ^)^ was applied to compute plate width at individual trabecular locations and characterize longitudinal and transverse trabeculae. Volumetric bone mineral density of transverse trabeculae (Tb.tBMD; mg/cc) was computed as the volumetric BMD in an ROI contributed by transverse trabeculae, while mean trabecular bone plate width (Tb.PW; μm) was computed by averaging individual trabecular plate widths over an ROI. Trabecular thickness (Tb.Th; μm) and trabecular spacing (Tb.Sp; μm) measures representing mean trabecular thickness and spacing between individual trabeculae, respectively, were computed using our star‐line‐based algorithm, which was previously demonstrated to yield accurate measures across resolution regimes covering ex vivo and in vivo imaging methods.^(^ ^36^ ^)^ The erosion index (EI) (no unit) was computed using our previously reported digital topological analysis algorithms,^(^ ^46^, ^47^ ^)^ except that fuzzy skeletonization was applied instead of binary skeletonization.^(^ ^48^ ^)^ The structure model index (SMI) (no unit) was computed using an algorithm proposed by Hildebrand and Rüegsegger.^(^ ^49^ ^)^ Specifically, a simulated thickening and differential analysis was applied on a triangulated surface of a structure to compute SMI as the surface area derivative with respect to the half‐thickness or radius. Validated CT‐based cortical bone segmentation algorithm^(^ ^42^ ^)^ was used to segment Cb regions and compute the Cb thickness (Cb.Th; mm) and Cb porosity (Cb.Poro; no unit). All Tb measures were computed at 4% to 6% distal tibial site over inner (60% peel region) and outer (the annular region between 30% and 60% peels) ROIs, while Cb measures were obtained at 14% to 16% tibial sites.

### Vertebral fracture reading

TLC chest CT scans at 5‐year follow‐up visits acquired under the COPDGene study protocol^(^ ^37^ ^)^ were used for morphological vertebral fracture reading. A team of six trained readers and two adjudicators reviewed CT scans to score vertebral fractures using AquariusNet (TeraRecon, Foster City, CA, USA) software. Readers identified central compression and wedge vertebral fractures on T_1_ to L_1_ vertebrae using the semiquantitative method of Genant.^(^ ^50^ ^)^ Only moderate or severe fractures by the Genant method were scored. A minimum of two readers using a three‐dimensional reconstruction recorded a score for each vertebra. Lack of agreement was adjudicated to a consensus score.

### Statistical data analyses

Descriptive statistics including means and standard deviations of micro‐CT‐ and CT‐derived Tb measures were calculated to examine value shifts of different measures using two different imaging modalities. Scatter plots were examined to develop the best approach to comparison of imaging results. Pearson's linear correlation of micro‐CT‐ (gold standard) and CT‐derived values of Tb measures over matching ROIs (*n* = 175) was computed to determine the strength of association between corresponding measures, and linear calibration equations for CT measures were developed. Intraclass correlation coefficients (ICC) of the values of individual measures from three repeat CT scans were computed to examine reproducibility of Cb and Tb measures (*n* = 15). To avoid additional interpolation‐related loss of image resolution and subsequent artifacts in bone measurements, matching ROIs were aligned for repeat CT scans or micro‐CT and CT images using appropriate registration transforms^(^ ^51^ ^)^ between the target images instead of applying those transforms on images.

Descriptive statistics including means and standard deviations for continuous variables and frequencies for categorical variables were calculated to characterize pilot study participants. Normal probability plots and tests showed no severe departure from normality for continuous variables representing potential risk factors included in analyses; there were a few high‐value outliers for CT outcomes. Student's *t* tests were used to compare unadjusted values of CT bone measures for groups of interest—participants with and without vertebral fracture and participants with and without COPD. Spearman correlation analysis was performed to investigate bi‐variable associations between CT bone outcomes and potential covariates for group comparison adjustment. Because of limited sample size, only variables that have been established as bone‐quality risk factors were tried out as possible covariates, including age, sex, body mass index (BMI), and COPD status for fracture groups' analysis. General linear models were used to compare groups of interest, taking into account the most important covariates. Results were verified using robust regression approach to exclude influence of outliers. Least squares (LS) means were calculated for groups with and without fracture, adjusting for COPD status as the most important covariate. Effect sizes were calculated for both unadjusted and adjusted group comparisons. SAS statistical software (version 9.4, SAS Institute, Inc., Cary, NC, USA) was utilized for analyses and a *p* value of <0.05 was considered statistically significant.

## Results

**Fig 3:**
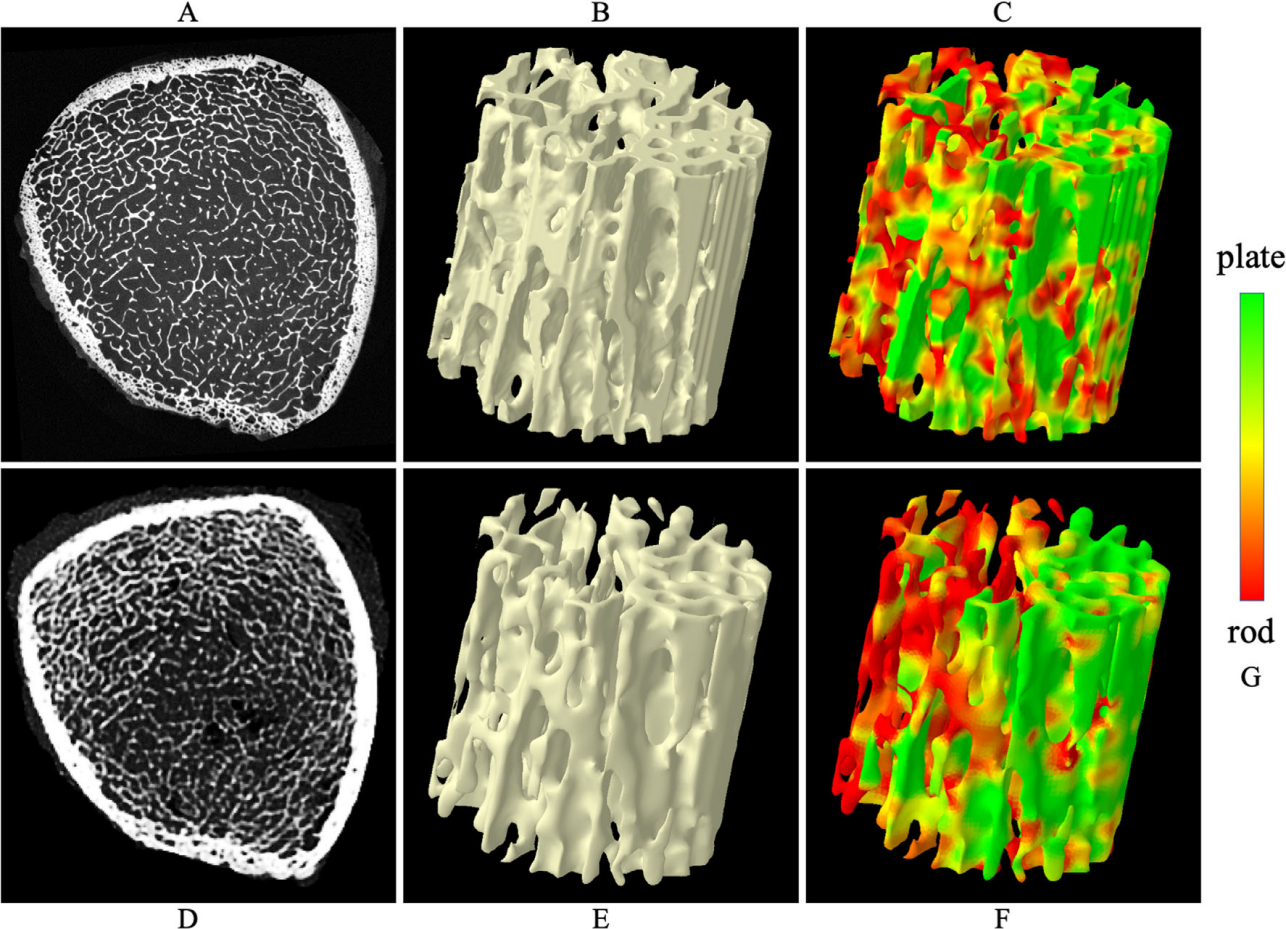
Comparison of trabecular bone (Tb) microstructure using micro‐CT and CT imaging. (*A*) An axial slice from micro‐CT image of a cadaveric ankle specimen. Circular field of view of micro‐CT imaging causes visible cortical bone (Cb) cropping in the axial view. (*B*) A 3D reconstruction of Tb micro‐network over a cylindrical region of interest (height: 7.65 mm; cross‐sectional diameter: 7.65 mm). (*C*) Color‐coded illustration of plate‐rod characterization of individual trabeculae. (*D*–*F*) Same as (*A*–*C*) but for matching locations from CT image of the same specimen. (*G*) The common color‐coding scale used in (*C*) and (*F*).

**Fig 4:**
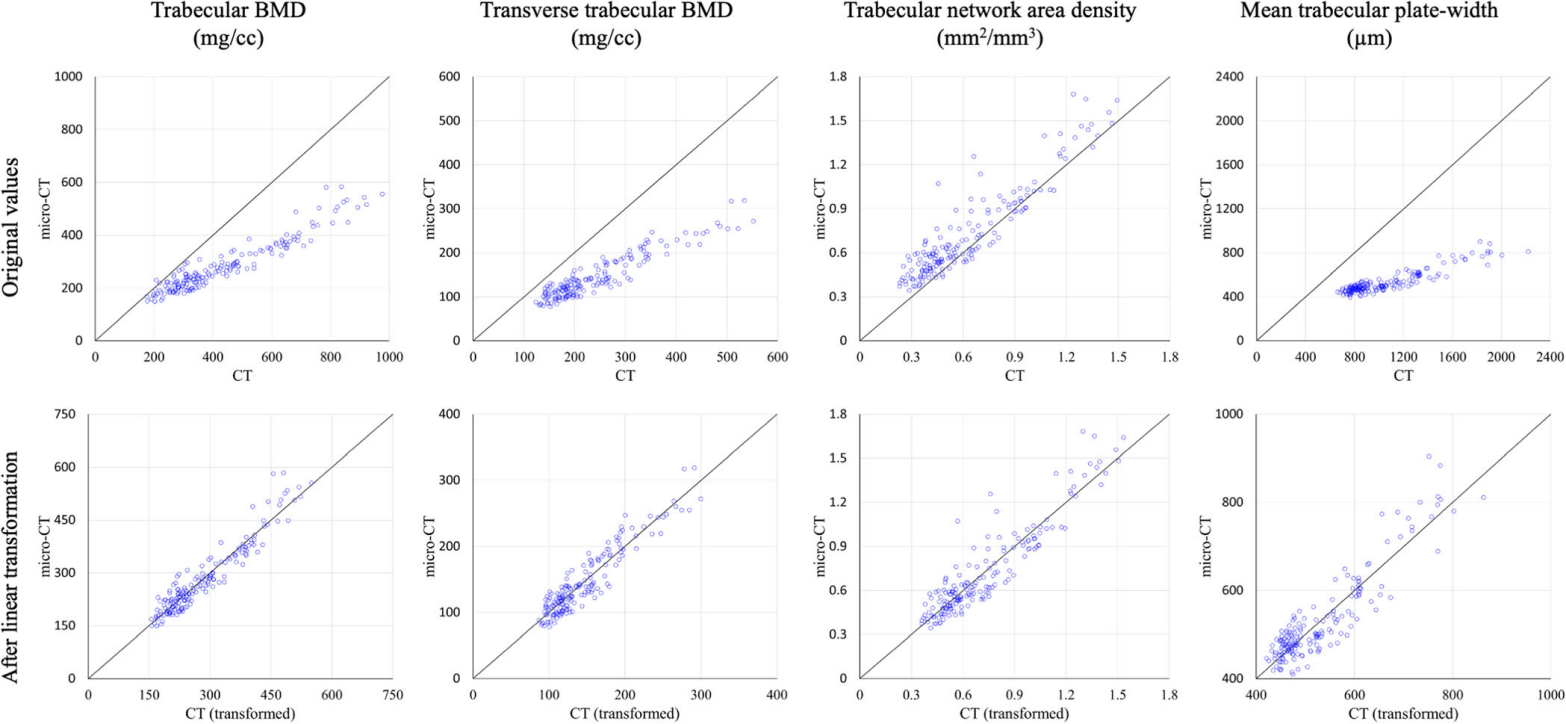
Correlation plots between micro‐CT‐ and CT‐derived trabecular bone microstructural measures before and after linear calibration. Identity lines are displayed in each plot for comparison. See Table 1 for linear calibration equations for different measures.

Tb microstructures and computerized characterization of Tb plates and rods using micro‐CT and CT imaging are visually compared in Fig. 3. Fig. 3*A*, *D* compare Cb and Tb on axial slices from matching locations in micro‐CT and CT images, while (*B*) and (*E*) show 3D reconstructions of Tb micro‐networks over matching ROIs. In general, blurring of both Cb and Tb microstructures in CT images is noticeable, causing partial loss of cortical pores and thinner trabeculae. However, agreement of relatively larger trabeculae in both 2D and 3D representation of the CT image with the matching micro‐CT image is noticeable. A similar trend is observed on computerized classification of trabecular plates and rods in (*C*) and (*F*). It is difficult to establish a correspondence of plate‐rod classification results at individual voxel or trabecular level, whereas agreements are noticeable over larger regions, eg, the left side of the ROI has more rods and the upper right side has more plates. Micro‐CT image was registered to CT image to generate matching locations for display purpose. Results of quantitative analyses comparing the results of micro‐CT‐ and CT‐derived Tb microstructural measurement are summarized in Table 1. In general, the observed values of individual Tb measures using micro‐CT and CT imaging are significantly different. However, high to moderate linear correlation of their values using micro‐CT and CT imaging was observed. For example, Tb volumetric BMD, network area, transverse trabeculae, and plate‐width measures (Tb.vBMD, Tb.NA, Tb.tBMD, Tb.PW) showed high correlation (*r* ∈ [0.901 0.935]) between their values using micro‐CT and CT imaging, whereas the correlation for SMI was moderate (*r* = 0.865). Tb thickness, spacing, and erosion index measures (Tb.Th, Tb.Sp, and EI) produced relatively low correlation (*r* ∈ [0.703 0.732]). To understand the relatively lower correlation (*r* = 0.901) of Tb.vBMD compared with that of Tb.NA (*r* = 0.924), we computed another measure by summing BMD values over thresholded bone microstructures and then dividing with the ROI volume. This bone density measure over Tb microstructures showed higher correlation (*r* = 0.952) between micro‐CT‐ and CT‐derived values with mean ± SD being 280.8 ± 97.0 and 430.0 ± 188.1 mg/cc, respectively. Linear calibration equation computed using simple regression analysis between micro‐CT‐ and CT‐derived values for different Tb measures are shown in Table 1. Correlation plots for micro‐CT‐ and CT‐derived values of different Tb measures before and after calibration are presented in Fig. 4.

**Table 1:** Mean and Standard Deviation of the Values of Tb Measures From Micro‐CT and CT Images of Cadaveric Ankle Specimens and Pearson Correlation Coefficients (*r*) and Linear Calibration Equations Among the Values of These Measures Over Matching ROIs (*n* = 175)

Variable	Micro‐CT mean (SD)	CT mean (SD)	Pearson correlation (*r*)	Calibration^a^ (CT to micro‐CT)	
				Intercept	Slope
Tb.vBMD (mg/cc)	505.9 (78.8)	1098.5 (61.7)	0.901	−757.679	1.150
Tb.tBMD (mg/cc)	144.6 (49.5)	240.0 (93.1)	0.935	25.384	0.497
Tb.NA (mm^2^/mm^3^)	0.72 (0.30)	0.62 (0.30)	0.924	0.145	0.931
Tb.PW (μm)	530.2 (99.9)	1045.7 (321.6)	0.913	233.730	0.284
Tb.Th (μm)	124.6 (14.1)	124.4 (16.9)	0.703	51.477	0.588
Tb.Sp (μm)	488.8 (93.8)	415.7 (108.0)	0.732	224.545	0.636
EI (no unit)	0.41 (0.18)	0.76 (0.46)	0.711	0.190	0.286
SMI (no unit)	0.69 (0.48)	1.64 (1.26)	0.865	0.154	0.325

Tb.vBMD = volumetric trabecular bone mineral density; Tb.tBMD = volumetric bone mineral density of transverse trabeculae; Tb.pBMD = volumetric bone mineral density of plate‐like trabeculae; Tb.NA = trabecular bone network area density; Tb.PW = mean trabecular bone plate width; Tb.Th = mean trabecular bone thickness; Tb.Sp = mean space between individual trabeculae; EI = trabecular bone erosion index; SMI = trabecular bone structure model index.

The *p* value was less than 0.0001 for all correlation results.

^a^ Simple linear regression analysis was used to derive calibration equations.

**Fig 5:**
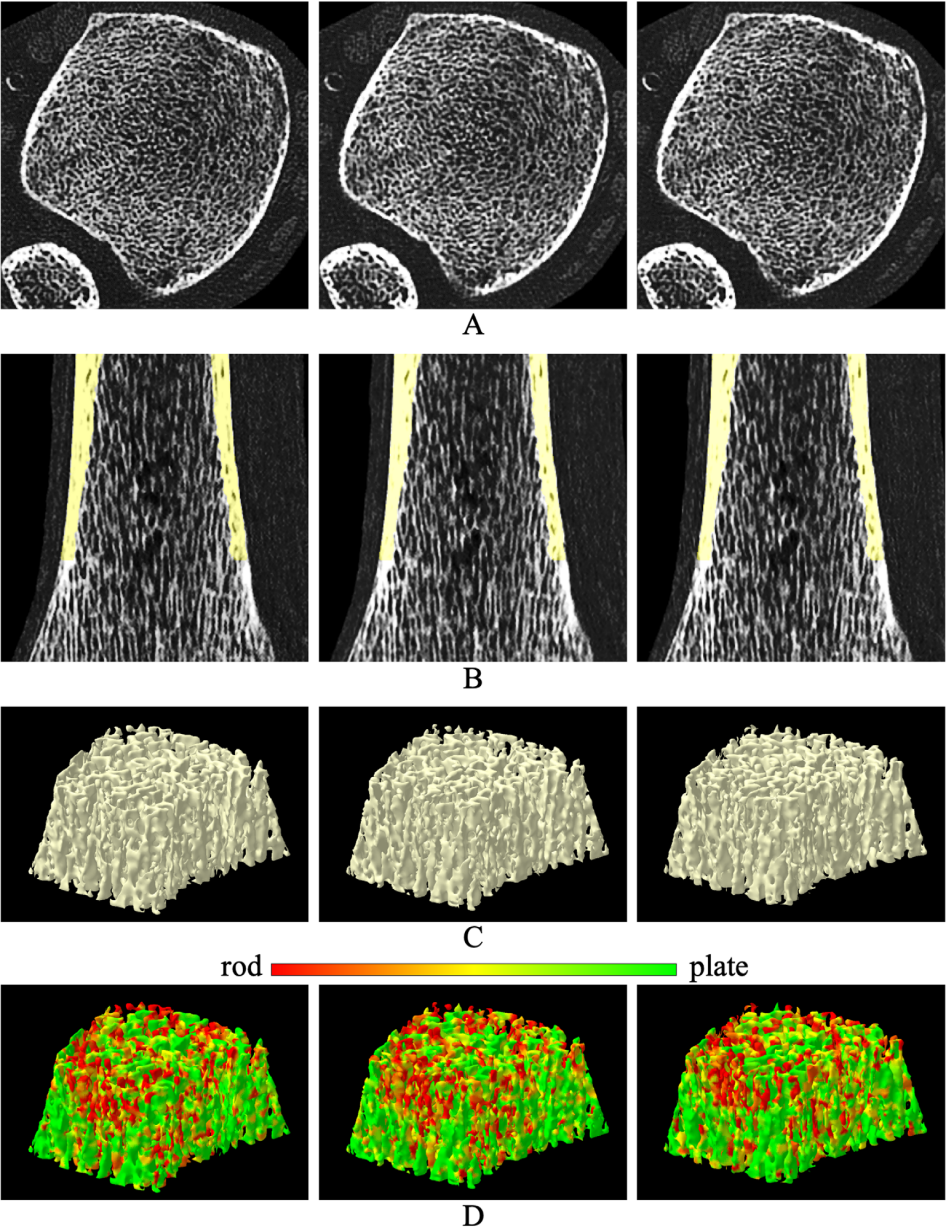
Reproducibility of CT‐based image and quantitative characterization of peripheral bone microstructure. Three columns represent the results from three repeat CT scans. (*A*, *B*) Axial and sagittal image slices from repeat scans of a cadaveric ankle specimen on a Siemens SOMMATOM Force CT scanner; segmented cortical bone (Cb) regions are overlaid on the sagittal slice. (*C*) Surface rendition of the trabecular bone (Tb) network at the inner region with 60% peel from the outer Cb surface. (*D*) Color‐coded displays of computed trabecular plate‐rod microstructure.

Repeat CT scan reproducibilities of bone microstructural imaging and computational characterization are illustrated in Fig. 5. For the display purpose, a post‐image registration algorithm was applied to match the two repeat CT scans to the initial scan of the specific specimen. Matching axial and sagittal image slices from the three CT scans are shown in (*A*) and (*B*). Cropping was applied to these images to highlight Tb microstructures inside the tibia. Segmented Cb region over 8% to 16% of distal tibia generated using our previously validated algorithm^(^ ^36^ ^)^ is overlaid on sagittal images in (*B*). Matching Tb microstructures as well as cortical pores in the initial and the two repeat CT scans are notable in both axial and sagittal images. 3D reconstructed Tb network as well as color‐coded displays of Tb plate‐rod characterization over matching ROIs from the three CT scans are presented in (*C*) and (*D*). In (*C*), matching trabeculae are notable in 3D renditions of Tb networks from the three scans. Also, in (*D*), the agreement of Tb plate‐rod characterization in three scans is prominent both at regional as well as individual trabecular levels. Reproducibility of Cb and Tb measures at 14% to 16% and 4% to 6% tibial sites (*n* = 15), respectively, are presented in Table 2. Also, reproducibilities of regional Tb measures over small matching ROIs (*n* = 225) are presented in the same table. Both Cb and Tb measures showed high reproducibility. Intra‐class correlation (ICC) for all Tb measures except erosion index was greater than 0.99 at both inner and outer ROIs; erosion index produced ICCs of 0.94 and 0.98 at inner and outer ROIs, respectively. ICCs for both Cb porosity and thickness measures were greater than 0.97. In general, reproducibility of regional Tb measures over small ROIs was slightly lower compared with the performance of corresponding measures over inner and outer ROIs at 4% to 6% distal tibia site. Tb volumetric BMD, transverse BMD, network area, plate‐width, and separation measures showed good regional reproducibility with ICC > 0.97. SMI produced an ICC of 0.88, while Tb thickness and erosion index showed ICC of 0.71 and 0.36, respectively.

**Table 2:** Repeat CT Scan Reproducibility of Cb Measures at 14% to 16% and Tb Measures at 4% to 6% Distal Tibial Site and Over Small Matching ROIs

Variable	ICC (95% CI)	ICC (95% CI)	ICC (95% CI)
Trabecular 4% to 6% site	Inner ROI (*n* = 15)	Outer ROI (*n* = 15)	Small ROI (*n* = 225)
Tb.vBMD (mg/cc)	0.994 (0.986, 0.998)	1.000 (0.999, 1.000)	0.981 (0.977, 0.985)
Tb.tBMD (mg/cc)	0.998 (0.995, 0.999)	0.999 (0.998, 1.000)	0.981 (0.977, 0.985)
Tb.NA (mm^2^/mm^3^)	0.999 (0.998, 1.000)	1.000 (1.000, 1.000)	0.990 (0.987, 0.992)
Tb.PW (μm)	0.999 (0.997, 1.000)	1.000 (1.000, 1.000)	0.978 (0.972, 0.982)
Tb.Th (μm)	0.997 (0.992, 0.999)	1.000 (0.999, 1.000)	0.709 (0.649, 0.761)
Tb.Sp (μm)	0.999 (0.997, 0.999)	0.998 (0.995, 0.999)	0.975 (0.969, 0.980)
EI (no unit)	0.943 (0.866, 0.976)	0.981 (0.953, 0.992)	0.357 (0.261, 0.446)
SMI (no unit)	0.997 (0.991, 0.999)	0.999 (0.998, 1.000)	0.884 (0.856, 0.908)
Cortical 14% to 16% site	(*n* = 15)		
Cb.Poro (no unit)	0.997 (0.992, 0.999)		
Cb.Th (mm)	0.975 (0.939, 0.990)		

ICC = intra‐class correlation; ROI = region of interest; Tb.vBMD = volumetric trabecular bone mineral density; Tb.tBMD = volumetric bone mineral density of transverse trabeculae; Tb.pBMD = volumetric bone mineral density of plate‐like trabeculae; Tb.NA = trabecular bone network area density; Tb.PW = mean trabecular bone plate width; Tb.Th = mean trabecular bone thickness; Tb.Sp = mean space between individual trabeculae; EI = trabecular bone erosion index; SMI = trabecular bone structure model index; Cb.Poro = mean cortical porosity; Cb.Th = mean cortical bone thickness.

At 4% to 6% distal tibial site, ICC of Tb measures were separately computed for inner and outer ROIs.

**Fig 6:**
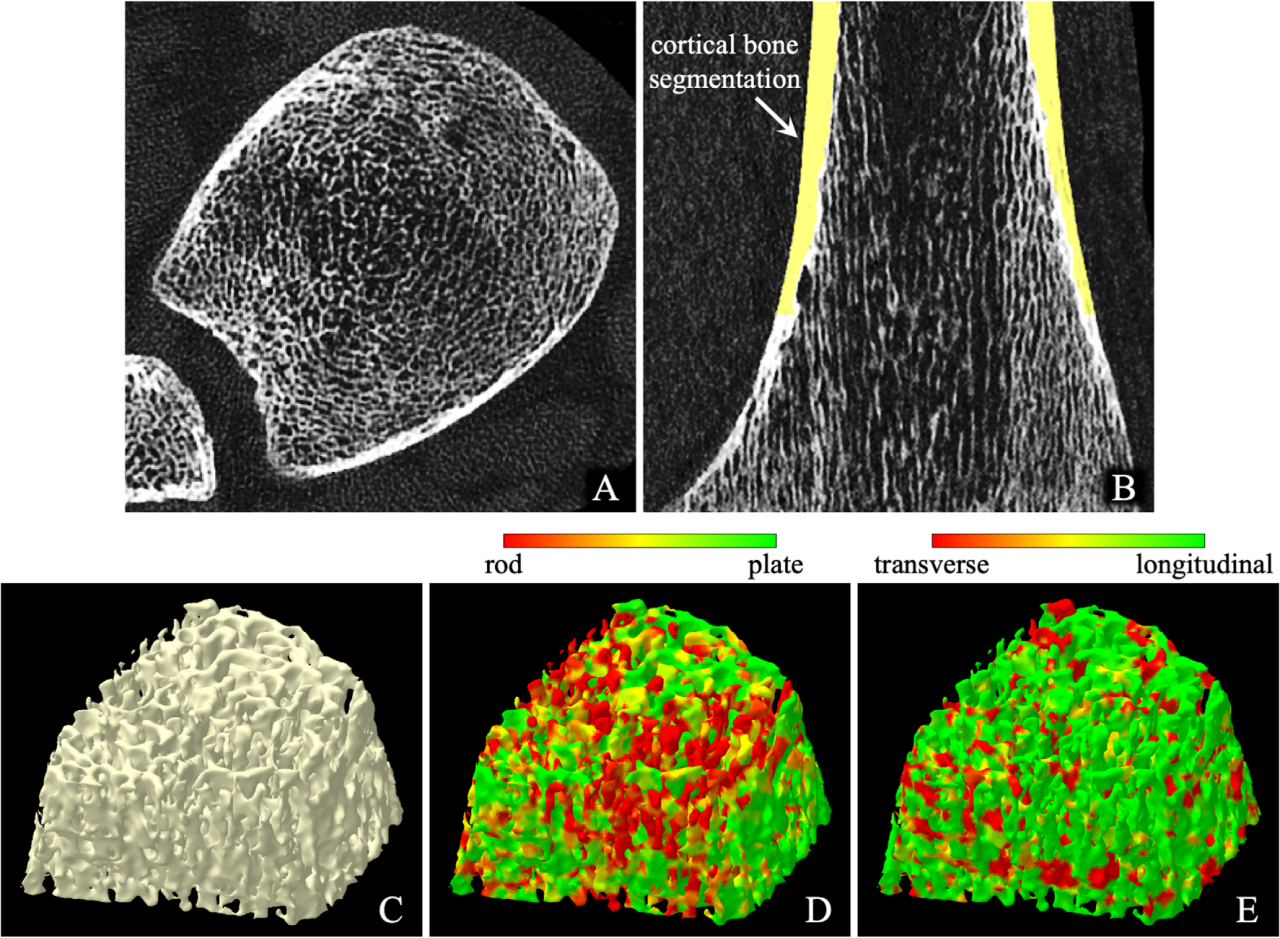
In vivo imaging of bone microstructure on a Siemens SOMMATOM Force CT scanner. (*A*, *B*) Axial and coronal image slices from a distal tibia CT scan. Cortical bone (Cb) segmentation over 8% to 16% of distal tibia is overlaid in (*B*). (*C*) Surface rendition of the Tb network at the inner region with 60% peel from the outer Cb surface. (*D*, *E*) Color‐coded displays of trabecular plate‐rod (*D*) and transverse‐longitudinal (*E*) microstructure.

Axial and coronal images of an in vivo CT distal tibia scan are shown in Fig. 6*A*, *B*, respectively. No visible marks of motion blur or beam‐hardening artifacts were observed in any of the 30 distal tibia CT scans acquired for this pilot study. All scans were successfully processed through fully automated and validated image processing protocols and algorithms developed in our laboratory,^(^ ^32^ ^)^ and expected Cb and Tb microstructural measures were successfully computed. Surface rendition of the 3D Tb microstructures and color‐coded displays of computed trabecular plate‐rod and transverse‐longitudinal classifications are shown in (*C–E*), respectively.

The 30 participants recruited for this pilot study are characterized in Table 3. All participants were white non‐Hispanic and 50% had COPD. Thirteen participants had GOLD status of 0, while the numbers of participants with GOLD status of 1, 2, 3, and 4 were 5, 7, 2, and 1, respectively; 2 participants were categorized as PRISm.^(^ ^52^, ^53^ ^)^ Participants were on average 70.5 ± 8.3 years old, and the study sample included slightly more females (53.7%). Three participants had history of non‐melanoma skin cancer, and 3 participants had history of other cancers (throat 1; larynx 1; and cervical 1). One participant had history inhaled corticosteroids use; no oral or systemic corticosteroid use was reported. No history of kidney disease was reported by any participant. By study design, all participants were current or former smokers (estimated pack‐years: 40.3 ± 22.3). Former smokers were identified based on their response to smoking‐related questionnaire at their 5‐year follow‐up COPDGene study visits, and the time elapsed since they quit smoking was 27.8 ± 12.7 years. Of the 19 participants classified as former smokers at the 5‐year follow‐up visit, 18 had quit smoking before their baseline visit. The COPD group included more heavy smokers (pack‐years: 47.6 ± 22.9) than the group without COPD (pack‐years: 32.9 ± 19.9). Although most of the participants (21/30, 70%) had at least one vertebral fracture, fractures were more prevalent in the participants with COPD than in those without COPD (80% versus 60%). No hip fracture was reported by any participant.

**Table 3:** Description of Pilot Study Participants Recruited From the University of Iowa COPDGene Cohort at 5‐Year Follow‐up Visits (*n* = 30)

Variable	*n* (%)
Females	16 (53.3%)
Race (white)	30 (100%)
Current smokers	11 (36.7%)
COPD	15 (50%)
Fracture 0	9 (30.0%)
1	10 (33.3%)
2 or more	11 (36.7%)
	Mean (SD)
Age (years)	70.53 (8.25)
Weight (kg)	80.52 (16.16)
Height (cm)	169.87 (10.03)
Body mass index (kg/m^2^)	27.80 (4.67)
Smoking (pack‐years)	40.3 (22.3)
6‐minute walk distance (ft)	1374.7 (329.8)

We expected that participants with vertebral fractures would have substantially lower quality of bone, specifically trabecular characteristics, because trabecular bone represents a high percent of vertebra bone. Power for two‐sample 2‐sided *t* test with alpha = 0.05 for our pilot study (*n* = 30 participants, unbalanced design 9/21 per group) calculated using SAS POWER procedure for different effect sizes shows power >0.8 for effect size 1.15 and higher (estimated power for effect size = 1 is 0.68; for effect size = 1.10 is 0.76). Post hoc power calculations based on *t* test degrees of freedom and achieved *p* values give similar estimates for our results (power >0.80 for tests with *p* < 0.01 and power 0.51 to 0.76 for *p* = 0.05 to 0.01).^(^ ^32^, ^54^ ^)^ We would expect somewhat reduced effect sizes after adjustment for COPD status.

Bi‐variable analysis of association (data not shown) between CT bone measures and age showed a negative association between bone outcomes and increasing age in outer region for Tb; specifically, Spearman correlation was −0.21 for plate width and −0.26 for trabecular thickness. For cortical bone, a correlation of 0.29 with porosity and −0.29 with thickness were found. Female sex was associated with worse Tb microstructure and the differences were relatively more significant in outer ROI. Females had lower Tb.tBMD (*p* < 0.1), higher Tb.Sp (*p* < 0.05), and lower cortical thickness (*p* < 0.001). BMI showed significant associations with several distal tibial bone measures. The observed Spearman correlations (*r* value) of BMI with Tb.tBMD, Tb.NA, Tb.PW, and Tb.Th at the inner ROI were 0.374 (*p* = 0.042), 0.397 (*p* = 0.30), 0.377 (*p* = 0.40), and 0.433 (*p* = 0.017), respectively. BMI and absence of COPD diagnosis were positively associated with bone measures, and these associations were strongest compared with the other factors that we investigated, including age, sex, weight, height, smoking quantity in pack‐years, and physical fitness based on the 6 minutes walking distance.

Analysis of bone measures for the three fracture groups showed that participants with one fracture and those with two or more fractures had very similar Tb characteristics, so these two groups were combined into one group for further analyses. Unadjusted comparisons for participants with and without fractures showed consistently better bone quality for participants without fracture (Table 4). The differences between groups were statistically significant (*p* < 0.05) for all Tb measures for inner ROI except thickness with high values for effect size (1 or above for most trabecular CT measures). Similar but somewhat weaker results appeared for outer ROI. Specifically, significant differences (*p* < 0.05) were observed between fracture and non‐fracture groups over outer ROIs for Tb.vBMD, Tb.tBMD, Tb.NA, and Tb.Sp measures (data not shown). Cortical bone porosity was significantly higher in the fracture group than in the non‐fracture group (0.20 versus 0.17, *p* < 0.05, effect size = 0.97). Adjustment for COPD status slightly decreased the estimated LS mean differences (Table 5), but the group differences were mostly statistically significant and effect sizes above 0.9. Validation with robust regression that reduces influence of outliers resulted in similar conclusions. Compared with former smokers at the baseline visit, a higher proportion of the pilot study participants who smoked at baseline had one or more fractures (81.8% versus 63.2%) or a COPD diagnosis (63.6% versus 42.1%), but these differences were not statistically significant. Fisher's exact test was used for comparison because of small sample size.

**Table 4:** Unadjusted CT Bone Outcomes Group Comparisons for Vertebral Fracture Status

Variable	No fracture (*n* = 9) mean (SD)	≥1 fracture (*n* = 21) mean (SD)	Mean difference (SD)	*t* Test *p* value	Effect size^a^
Trabecular 4% to 6% site (inner: 60% peel)					
Tb.vBMD (mg/cc)	1135.4 (48.3)	1088.6 (32.0)	46.9 (37.4)	0.004	1.25
Tb.tBMD (mg/cc)	301.0 (93.6)	201.0 (61.4)	99.9 (72.1)	0.002	1.39
Tb.NA (mm^2^/mm^3^)	0.77 (0.30)	0.48 (0.17)	0.29 (0.21)	0.002	1.36
Tb.PW (μm)	1170.7 (212.4)	968.2 (143.7)	202.5 (166.3)	0.005	1.22
Tb.Th (μm)	126.2 (14.7)	116.6 (6.5)	9.5 (9.6)	0.019	0.99
Tb.Sp (μm)	331.3 (79.5)	499.9 (187.7)	−168.6 (164.2)	0.016	−1.03
EI (no unit)	0.70 (0.17)	0.86 (0.37)	−0.16 (0.33)	0.232	−0.49
SMI (no unit)	1.54 (0.57)	1.85 (1.05)	−0.31 (0.94)	0.414	−0.33
Cortical 14% to 16% site					
Cb.Poro (no unit)	0.17 (0.01)	0.20 (0.03)	−0.03 (0.03)	0.022	−0.97
Cb.Th (mm)	1.64 (0.60)	1.56 (0.59)	0.08 (0.59)	0.738	0.13

Tb.vBMD = volumetric trabecular bone mineral density; Tb.tBMD = volumetric bone mineral density of transverse trabeculae; Tb.pBMD = volumetric bone mineral density of plate‐like trabeculae; Tb.NA = trabecular bone network area density; Tb.PW = mean trabecular bone plate width; Tb.Th = mean trabecular bone thickness; Tb.Sp = mean space between individual trabeculae; EI = trabecular bone erosion index; SMI = trabecular bone structure model index; Cb.Poro = mean cortical porosity; Cb.Th = mean cortical bone thickness.

^a^ Effect size was calculated as (mean difference)/(pooled standard deviation).

**Table 5:** Adjusted CT Bone Outcomes Group Comparisons for Vertebral Fracture Status (Least Squares Means Adjusted for COPD Status Were Calculated)

Variable	No fracture (*n* = 9) LS mean	≥1 fracture (*n* = 21) LS mean	LS mean difference (SE)	*t* Test *p* value	Effect size^a^
Trabecular 4% to 6% site (inner: 60% peel)					
Tb.vBMD (mg/cc)	1131.25	1090.36	40.90 (14.64)	0.010	1.14
Tb.tBMD (mg/cc)	293.24	204.37	88.87 (28.35)	0.004	1.28
Tb.NA (mm^2^/mm^3^)	0.74	0.49	0.25 (0.08)	0.005	1.26
Tb.PW (μm)	1150.79	976.72	174.1 (64.43)	0.0118	1.10
Tb.Th (μm)	125.06	117.12	7.95 (3.74)	0.043	0.87
Tb.Sp (μm)	344.00	494.44	−150.43 (66.37)	0.032	−0.93
EI (no unit)	0.70	0.86	−0.16 (0.14)	0.252	−0.48
SMI (no unit)	1.57	1.84	−0.27 (0.39)	0.494	−0.28
Cortical 14% to 16% site					
Cb.Poro (no unit)	0.17	0.20	−0.02 (0.01)	0.0333	−0.92
Cb.Th (mm)	1.60	1.57	0.03 (0.24)	0.908	0.05

LS = least squares; Tb.vBMD = volumetric trabecular bone mineral density; Tb.tBMD = volumetric bone mineral density of transverse trabeculae; Tb.pBMD = volumetric bone mineral density of plate‐like trabeculae; Tb.NA = trabecular bone network area density; Tb.PW = mean trabecular bone plate width; Tb.Th = mean trabecular bone thickness; Tb.Sp = mean space between individual trabeculae; EI = trabecular bone erosion index; SMI = trabecular bone structure model index; Cb.Poro = mean cortical porosity; Cb.Th = mean cortical bone thickness.

^a^ Effect size was calculated as the (LS means difference)/√MSE, where MSE = the mean square error from general linear model.

## Discussion

Large differences in CT‐ and micro‐CT‐derived values of different Tb measures suggest that CT‐derived values are not directly comparable with their gold standard measures. However, high to moderate linear correlation of CT‐derived values with their micro‐CT‐derived values suggests that CT‐derived measures are comparable with their gold standard values after calibration (Fig. 4). Thus, CT imaging offers surrogate measures of Tb density and microstructure, and proper calibration may be needed when different scanners or imaging modalities are used in cross‐sectional and longitudinal studies examining response to different disease progression and intervention. High correlation (*r* = 0.952) of BMD over segmented Tb microstructure further assures the feasibility of CT for Tb microstructural imaging. Relatively lower correlation (*r* = 0.901) of the Tb.vBMD measure computed as average density over an entire ROI may be caused by distortions over the marrow space during specimen preparation and thawing for micro‐CT imaging. Experimental results suggest that CT‐derived measures of Tb spacing, thickness, and erosion index are less reliable because of their low correlation (*r* ∈ [0.703 0.732]) with micro‐CT‐derived values.

Also, experimental results demonstrate that CT‐based measures of Cb and Tb microstructure are highly reproducible, confirming the robustness of CT‐based imaging of peripheral bone microstructure and the analytic methods developed in our laboratory. In particular, the strong reproducibility of Tb measures related to volumetric BMD, network area, transverse microstructure, plate‐rod distribution, and spacing over small ROIs suggests that this method is suitable to detect and study heterogenous bone alterations and their impact on fracture risk. Tb erosion index and thickness measures showed low regional reproducibility, while that of SMI was found to be moderate.

MRI and HR‐pQCT imaging have been widely used for bone microstructural measurements in human studies.^(^ ^8^, ^25^, ^26^, ^27^, ^28^, ^29^, ^30^ ^)^ However, the use of whole‐body clinical CT for in vivo bone microstructural analysis is relatively recent.^(^ ^32^ ^)^ Although the spatial resolution achievable by emerging CT scanners is inferior to HR‐pQCT technology, it allows segmentation and assessment of bone microstructure at peripheral sites (eg, ankle, wrist, or knee) using a relatively low radiation dose, and previous studies have demonstrated a strong association between CT‐based bone microstructural measures with their micro‐CT‐derived values and experimental bone strength.^(^ ^32^ ^)^ Moreover, CT imaging has several advantages compared with HR‐pQCT. Modern CT scanners are capable of imaging 10 cm of scan length at a UHR mode in just 6 seconds compared with ~2.9 minutes for 0.9 cm of scan length using HR‐pQCT,^(^ ^55^ ^)^ thus reducing motion blur artifacts. Additionally, emerging CT technology offers major radiation dose reduction, while simultaneously increasing spatial resolution and signal‐to‐noise ratio (SNR).^(^ ^56^ ^)^ Also, whole‐body CT allows bone measurement at central sites, eg, spine and hip. If CT is established as effective for quantitative bone microstructural imaging, its wide availability in clinical environments and low radiation will immediately put it as a front‐runner for large multicenter musculoskeletal studies. However, a major challenge of CT‐based bone microstructural imaging is that scanners from different vendors and those from the same vendor but of different models are often associated with different spatial resolution or sharpness features, which may affect the measured values of bone microstructure. Therefore, researchers should examine between‐scanner correlation and calibration factors before designing multicenter bone imaging studies using CT scanners.^(^ ^32^ ^)^

This pilot study shows that among all the risk factors investigated, BMI was the strongest predictor of CT‐based bone measures within a group of smokers with and without lung disease. The association between BMI and osteoporosis is well known.^(^ ^57^, ^58^ ^)^ In a study of 1765 elderly men and women, Nguyen and colleagues^(^ ^57^ ^)^ observed that, compared with subjects whose BMI was less than 27 kg/m^2^, those with BMI values greater than 27 kg/m^2^ had higher age‐adjusted BMD (8% in men and 10% in women). In another cross‐sectional study of 505 women aged 50 to 84 years, Asomaning and colleagues^(^ ^58^ ^)^ found that, after adjustment for age, prior hormone replacement therapy (HRT) use, and other factors, women's odds ratios of osteoporosis versus osteopenia/normal BMD were 1.8 for underweight (<19 kg/m^2^), 0.46 for overweight (BMI 25 to <30 kg/m^2^), and 0.22 for obese (≥30 kg/m^2^) women when compared with women of normal BMI.

Results comparing bone microstructure between groups based on vertebral fracture status show statistically significant differences for all Tb microstructural measures at the inner ROI with 60% peel except for thickness, EI, and SMI. The true resolution of the CT scanner (201.6 μm in‐plane and 238.1 μm *z*‐axis resolution) used for this pilot study is larger than the thickness of human trabecula resulting inherently fuzzy representation of Tb microstructure in acquired images. Relatively low accuracy of Tb thickness measures using a similar CT scanner, determined in terms of linear correlation (*r* = 0.71) with micro‐CT‐based measures, was observed in a previous study.^(^ ^32^ ^)^ The low accuracy of the Tb thickness derived using the current CT scanner may be due to the limited resolution of CT imaging. Like the Tb thickness measure, it was experimentally observed that both EI and SMI measures have limited accuracy at CT imaging resolution in terms of their correlation with micro‐CT‐derived values and bone strength.^(^ ^32^ ^)^ The statistically significant difference in Tb plate width between the fracture and non‐fracture groups is in agreement with observations of several histologic studies,^(^ ^11^, ^12^, ^18^ ^)^ and the relationships between erosion of trabecular plates to rods and higher fracture prevalence have been further confirmed in this pilot study. Tb.tBMD is a unique measure computed using our previously validated tensor scale analysis algorithm,^(^ ^45^ ^)^ which represents the density of transverse trabeculae. The results suggest that participants with any vertebral fracture have fewer transverse trabeculae compared with those without fractures. This observation validates the mechanical modeling results,^(^ ^59^ ^)^ suggesting that loss of transverse trabeculae is associated with a marked reduction in bone strength, leading to failure due to buckling of longitudinal trabeculae. Also, results in Table 4 suggest that participants in the fracture group have reduced trabeculae, measured in terms of Tb.NA, with increased Tb spacing compared with participants in the non‐fracture group. A similar but weaker group difference of Tb measures (data not provided) were observed at outer Tb region between 30% and 60% peel from the outer cortical bone surface. There were no statistically significant differences in Cb thickness and porosity measures between fracture and non‐fracture groups. As shown in Table 5, differences of Tb microstructural measures between fracture and non‐fracture groups, excluding Tb thickness, EI, and SMI, remain statistically significant after adjusting for age, BMI, and COPD status. Similar results were observed when adjusted for sex, age, and then COPD status. The data observed in this pilot study suggest that the relationships between bone microstructure and vertebral fractures are more intrinsic, and these relationships remain unperturbed across sex, age, BMI, and COPD status. Comparative analyses of bone measures for groups with different numbers of vertebral fractures showed that participants with fractures have very similar Tb microstructural characteristics, irrespective of the number of prevalent fractures. This observation of our pilot study is in line with published literature that history of previous osteoporotic fracture is a strong risk factor for subsequent fractures.^(^ ^60^, ^61^, ^62^ ^)^

Fifty percent of participants in this pilot study had COPD as assessed by spirometry. Osteoporosis is a major comorbidity of COPD,^(^ ^63^ ^)^ and the prevalence of osteoporotic fractures among patients with COPD is higher than the normal population.^(^ ^64^ ^)^ The data presented here demonstrate that CT‐based methods of measuring Cb and Tb microstructure are robust and suitable for in vivo studies evaluating effects of different risk factors on bone microstructural quality and their implications on bone strength and fracture risk. The population presented here is part of a larger, NIH‐funded study examining the impact of COPD‐related factors on bone microstructure and their implications for fracture risk in a larger cohort.

Smoking is a known risk factor for bone loss and fractures, and it appears to be independent of other risk factors such as advanced age, BMI, sex, and gonadal status.^(^ ^65^, ^66^ ^)^ The Dubbo Osteoporosis Epidemiologic Study (DOES), a longitudinal study of men and women over the age of 60 years, found that smoking is associated with lower BMD at the femoral neck and spine, independent of calcium intake and body weight.^(^ ^67^ ^)^ A Norwegian longitudinal study of 34,856 adults ≥50 years of age found an increased risk of hip fracture for both female (risk ratio [RR] = 1.5 and 95% confidence interval [CI] 1.0–2.4) and male smokers (RR = 1.8 and 95% CI 1.2–2.9) compared with non‐smokers.^(^ ^68^ ^)^ In a study involving 58 white women with vertebral crush fractures and 58 age‐matched healthy white women with no history of osteoporosis, Aloia and colleagues^(^ ^69^ ^)^ observed that smoking was more prevalent among women with fractures compared with controls. In this pilot study, a higher proportion of participants who smoked at or beyond their baseline visit had vertebral fractures compared with participants who quit smoking before their baseline visit. However, this difference was not statistically significant, and a larger sample size is required to understand the relationship between smoking and bone microstructure and its impact on fracture risk.

There are a few limitations of this pilot study. Participants were all white, limiting the generalizability of our findings to other races and ethnicities.^(^ ^70^ ^)^ The study population is older, and thus the observations will need to be extended by future studies in a younger group. Additionally, the COPDGene Iowa Cohort represents a rural population, and the results may not be applicable to an urban population with a different lifestyle.^(^ ^71^ ^)^ Finally, the sample size of this pilot study is small, although findings are statistically significant. The small sample size of this pilot study prohibits fully adjusted group comparisons.

Experimental results show that quantitative methods using emerging CT imaging for Cb and Tb microstructural measures are reproducible. The results observed in our pilot human study are novel as they demonstrate the differences in CT‐based cortical and trabecular bone microstructures among smokers with and without vertebral fractures. The pilot study also demonstrates the feasibility of emerging whole‐body CT‐based methods for evaluating disease effects on cortical and trabecular bone microstructures and their impact on fracture risk.

## Disclosures

All authors state that they have no conflicts of interest.

## Author contributions

**Xiaoliu Zhang:** Conceptualization; methodology; software; validation; visualization; writing‐original draft; writing‐review & editing. **Alejandro Comellas:** Conceptualization; methodology; resources; writing‐review & editing. **Elizabeth Regan:** Conceptualization; methodology; writing‐review & editing. **Indranil Guha:** Methodology; writing‐review & editing. **Amal Shibli‐Rahhal:** Conceptualization; methodology; writing‐review & editing. **Mishaela Rubin:** Conceptualization; methodology; writing‐review & editing. **Paul DiCamillo:** Conceptualization; methodology; writing‐review & editing. **Elena Letuchy:** Data curation; formal analysis; validation; writing‐original draft; writing‐review & editing. **R Graham Barr:** Conceptualization; methodology; writing‐review & editing. **Eric Hoffman:** Conceptualization; methodology; resources; writing‐review & editing. **Punam Saha:** Conceptualization; data curation; formal analysis; funding acquisition; investigation; methodology; project administration; resources; supervision; validation; visualization; writing‐original draft; writing‐review & editing.

### Peer review

The peer review history for this article is available at https://publons.com/publon/10.1002/jbm4.10484.
